# *Cordyceps militaris* Modulates Intestinal Barrier Function and Gut Microbiota in a Pig Model

**DOI:** 10.3389/fmicb.2022.810230

**Published:** 2022-03-17

**Authors:** Hongmei Zheng, Haigang Cao, Deming Zhang, Jiahe Huang, Jinshu Li, Shaoying Wang, Junfeng Lu, Xiao Li, Gongshe Yang, Xin’e Shi

**Affiliations:** Key Laboratory of Animal Genetics, Breeding and Reproduction of Shaanxi Province, Laboratory of Animal Fat Deposition and Muscle Development, College of Animal Science and Technology, Northwest A&F University, Yangling, China

**Keywords:** *Cordyceps militaris*, intestinal barrier, gut microbiota, immunity, pig

## Abstract

This study investigated the effects of *Cordyceps militaris* (CM) on intestinal barrier function and gut microbiota in a pig model. A total of 160 pigs were randomly allocated to either a control group (fed the basal diet) or a CM group (fed the basal diet supplemented with 300 mg/kg CM). CM improved intestinal morphology and increased the numbers of goblet cells and intraepithelial lymphocytes. CM also elevated the expression of zona occluden-1, claudin-1, mucin-2 and secretory immunoglobulin A. Furthermore, the mucosal levels of pro-inflammatory cytokines were downregulated while the levels of anti-inflammatory cytokines were upregulated in the CM group. Mechanistically, CM downregulated the expression of key proteins of the TLR4/MyD88/NF-κB signaling pathway. Moreover, CM altered the colonic microbial composition and increased the concentrations of acetate and butyrate. In conclusion, CM can modulate the intestinal barrier function and gut microbiota, which may provide a new strategy for improving intestinal health.

## Introduction

The intestine is not only the largest digestion and absorption organ but also the largest compartment of the immune system because it provides a mucosal barrier and various microbiota to promote body health (Donaldson et al., 2016). The intestinal mucosal barrier can prevent the invasion of pathogens and food-borne antigens (Luissint et al., 2016). It is a complex structure composed of the following three barriers: physical barrier, biochemical barrier, and immunological barrier. The physical barrier refers to intestinal epithelial cells (IECs), and the junction complexes include tight junctions, adherens junctions, desmosomes, and gap junctions (Peterson and Artis, 2014). Tight junction proteins (TJPs) can prevent potentially harmful substances or pathogens from entering the body by regulating the permeability of the barrier (Ulluwishewa et al., 2011). The biochemical barrier is mainly composed of mucin glycoproteins, antimicrobial peptides and secretory immunoglobulin A (SIgA), which can prevent epithelial cells from directly contacting bacteria and is essential for disease prevention (Gill et al., 2011). The immunological barrier is composed of gut-associated lymphoid tissue (GALT), immune cells and related cytokines. Cytokines affect the integrity of the intestinal barrier by regulating inflammation (Luissint et al., 2016). Moreover, the intestinal epithelium can express innate immunity by regulating molecular Toll-like receptors (TLRs) (Rakoff-Nahoum et al., 2004). Thus, regulating the mucosal barrier function can promote intestinal health (Vancamelbeke and Vermeire, 2017).

Gut microbiota have been demonstrated to produce bacteriocins and short-chain fatty acids (SCFAs), and inhibit pathogens thus regulating intestinal health (Flint et al., 2012; Valdes et al., 2018). Differences in microbial community diversity, composition and function are related to intestinal diseases (Lozupone et al., 2012). In addition, SCFAs, such as acetate, propionate, and butyrate, are ensemble products of intestinal microbial fermentation and play a crucial role in enhancing intestinal barrier function and maintaining mucosal immune function (Nicholson et al., 2012; Rooks and Garrett, 2016). A related study has shown that gastric infusion of SCFAs could improve piglet intestinal morphology and maintain intestinal barrier function (Diao et al., 2019). Thus, improving intestinal microbial composition has become a new biological target to promote intestinal health.

It is generally accepted that diet has a major effect on intestinal function and gut microbiota (Makki et al., 2018; Adebowale et al., 2019), and increasing attention is focused on functional food research. Therefore, supplementing functional foods in the diet is a promising strategy to improve intestinal function and gut microbiota (Wan et al., 2019). *Cordyceps militaris* (CM), which belongs to the class *Ascomycetesis*, is widely used as a health products (Kwon et al., 2018). CM possess multi-functions of enhancing immunity, anti-inflammatory and antioxidant with fewer adverse effects, foe it’s enriched in active ingredients such as cordyceps polysaccharide, D-mannitol, and cordycepin, etc. (Wang et al., 2012; Seo et al., 2018). Currently, artificial culture of CM has greatly increased its production and lowered its cost (Dong et al., 2015; Lou et al., 2019). CM has been demonstrated to enhance immunity by regulating immune cells and cytokines (Sun et al., 2014; Lee et al., 2015). In addition, the hybrid antimicrobial peptide magainin II-cecropin B gene was transformed into medicinal CM, which maintained the integrity of the intestinal mucosal barrier by upregulating TJPs (Zhang et al., 2018). Interestingly, CM also affected intestinal health by altering the composition of gut microbiota in humans (Gamage et al., 2018). However, research on the effects of CM on intestinal barrier function and gut microbiota in pigs is limited.

The aim of the present study was to investigate the effects of CM on intestinal barrier function and gut microbiota, as well as the underlying regulatory mechanisms. Since the gastrointestinal organs of pigs are closer to humans, we used pigs as the model for studying the role of CM in intestinal pathophysiology and related indicators of the physical barrier, biochemical barrier, immunological barrier, and gut microbiota. Our study will be beneficial to the development and application of CM as a potential strategy for improving intestinal health.

## Materials and Methods

### Ethics Statement

All animal protocols were approved and performed according to the guidelines of the Animal Welfare Committee of Northwest A&F University (Yangling, Shaanxi, China) (approval number: 20190603–027).

### Experimental Design and Sample Collection

A total of 160 pigs (Duroc × [Landrace × Yorkshire], 36.82 ± 0.20 kg, half female and half castrated male) were selected and assigned to two groups. Each group consisted of 80 pigs and they were housed in five pens, with sixteen pigs per pen. One group was fed a basal diet (Control group), and the other group was fed a basal diet supplemented with CM at 300 mg/kg (CM group). All pigs had free access to feed and water throughout the experimental period. Feed intake was recorded daily. The composition and nutrient content of the diets are shown in Table 1. CM was obtained from Shaanxi Xinli Biotechnology Co., Ltd. (Hanzhong, China) and its main active ingredients and nutritional composition (Table 2) were determined in the testing organization (Qingdao Kechuang Quality Testing Co., Ltd., Qingdao, China). Additionally, the dose of CM was determined on the basis of a previous study showing that pigs fed 300 mg/kg CM were comparable to pigs fed carbadox (Richert et al., 2019). All pigs were housed in an environmentally controlled room and had free access to feed and clean water. The feeding experiment lasted for 88 days from 102 to 190 days old.

**TABLE 1:** Ingredients and nutrient analysis of experimental diets.

Items	102–153 days		154–190 days	
	Control	CM	Control	CM
Ingredient,%				
Corn	75.28	75.25	78.91	78.88
Soybean meal	21.1	21.1	16.10	16.10
Wheat bran	–	–	1.80	1.80
Soybean oil	0.39	0.39	–	–
Calcium monophosphate	0.64	0.64	0.52	0.52
Stone powder	0.88	0.88	0.85	0.85
Vitamins and minerals Premix*^a^*	0.60	0.60	0.60	0.60
NaCl	0.33	0.33	0.33	0.33
Lysine sulfate (70%)	0.52	0.52	0.58	0.58
Threonine (98%)	0.09	0.09	0.14	0.14
Methionine (98%)	0.11	0.11	0.10	0.10
Choline chloride (60%)	0.05	0.05	0.05	0.05
Tryptophan (98%)	0.01	0.01	0.02	0.02
Cordyceps militaris powder	–	0.03	–	0.03
Total	100	100	100	100
Nutrient component*^b^*,%				
Dry matter	89.41	90.56	12.15	11.93
Crude protein	16.37	15.79	13.79	13.74
Crude fiber	2.30	2.30	2.30	2.30
Crude ash	3.56	3.63	0.53	0.55
Calcium	0.61	0.52	3.51	3.62
Total phosphorus	0.48	0.40	0.37	0.5
Available phosphorus	0.18	0.18	0.14	0.14
NE, MJ/kg	10.25	10.25	10.25	10.25

*^a^Premix supplied per kilogram of meal: vitamin A, 6,500 IU; vitamin D_3_, 2,850 IU; vitamin E, 30 mg; vitamin K_3_, 2 mg; vitamin B_1_, 40 mg; vitamin B_2_, 5 mg; vitamin B_6_, 3 mg; vitamin B_12_, 0.03 mg; niacin, 25 mg; vitamin C, 250 mg; calcium pantothenate, 9 mg; folic acid, 1 mg; biotin, 0.3 mg; choline chloride, 300 mg; Zn, 100 mg; Fe, 200 mg; Cu, 90 mg; Mn, 25 mg; I, 0.35 mg; Se, 0.35 mg; P, 0.1%; NaCl, 0.4%; lysine, 0.1%; Ca, 0.9%. –, representative not added.*

*^b^Nutrient contents are calculated values.*

**TABLE 2:** Composition of CM.

Active ingredient	Content (%)	Nutritional components	Content (%)
Cordyceps Polysaccharide	4.96	Crude protein	15.97
D-Mannitol	3.75	Crude fat	2.79
Total sterols	0.60	Crude ash	3.18
Cordycepin	0.16	Calcium	0.15
Adenosine	0.04	Phosphorus	0.61

### Sample Collection

Two pigs (one female and one castrated male) from each pen were selected according to average body weight and slaughtered after an overnight fast. The duodenum tissues were washed with sterile phosphate-buffered saline (PBS). Then, some of the duodenal samples were fixed in 4% paraformaldehyde solution to study the morphological changes. After collecting mucosal samples, the other duodenal samples were put in liquid nitrogen to freeze and transferred to a −80°C refrigerator for storage. Colonic digesta were collected into sterile tubes and kept in liquid nitrogen for analysis of gut microbiota and SCFAs. Then, colonic digesta pH was measured by a portable pH meter (Oakton Instruments, Illinois, United States). All samples used for RNA extraction and protein separation were stored at −80°C, and the samples of two pigs in each pen were measured after mixing.

### Assessments of Duodenal Morphology and Cell Count

Assessments of duodenal morphology and cell count were conducted according to a previous study (Zhu et al., 2013). Briefly, the paraffin sections were stained with hematoxylin-eosin (H&E) or periodic acid Schiff (PAS) according to standard histological methods. The villus height and crypt depth were evaluated by measuring at least five different regions in each section using a fluorescence microscope (LECIA DM6 B, Lecia, Germany) with ImageJ software. The number of intraepithelial lymphocytes (IELs) and goblet cells were counted by analyzing five different villi in each section. The results were expressed as the number of IELs or goblet cells per 100 epithelial cells.

### Secretoryn Immunoglobulin A and Cytokine Analysis in the Duodenal Mucosa

The duodenal mucosal tissue was added to PBS (9 mL per gram of tissue) for homogenization. The homogenate was centrifuged at 2,500 × *g* for 20 min, and the supernatant was used to determine the concentrations of SIgA and cytokines. The concentrations of SIgA, interferon-γ (IFN-γ), tumor necrosis factor α (TNF-α), interleukin-10 (IL-10), interleukin-4 (IL-4), interleukin-2 (IL-2), and interleukin-12 (IL-12) were determined with a commercial ELISA kit (Hengyuan Biotechnology Co., Ltd., Shanghai, China). All procedures were performed in accordance with the instructions of the manufacturer.

### Real-Time Quantitative Polymerase Chain Reaction

Total RNA was extracted from duodenal tissue, reverse transcription, and Real-Time Quantitative Polymerase Chain Reaction (RT-qPCR) were performed as previously described (Shi et al., 2019). Briefly, the results were calculated according to the expression of β-actin with the 2^–ΔΔ*Ct*^ method. The primer sequences for genes are listed in Table 3.

**TABLE 3:** Primers sequences for RT-qPCR.

Genes	Primer sequence (5′–3′)	Product length (bp)	GenBank accession
*ZO-1*	F: CAGCCCCCGTACATGGAGA R: GCGCAGACGGTGTTCATAGTT	114	XM_021098896.1
*Occludin*	F: CTACTCGTCCAACGGGAAAG R: ACGCCTCCAAGTTACCACTG	158	NM_001163647.2
*claudin-1*	F: GCCACAGCAAGGTATGGTAAC R: AGTAGGGCACCTCCCAGAAG	140	NM_001244539.1
*MUC-2*	F: CTGCTCCGGGTCCTGTGGGA R: CCCGCTGGCTGGTGCGATAC	101	XM_021082584.1
*TLR4*	F: ATATGGCAGAGGTGAAAGCAC R: GAAGGCAGAGATGAAAAGGGG	125	NM_001113039.2
*MyD88*	F: TGGTGGTGGTTGTCTCTGATGA R: TGGAGAGAGGCTGAGTGCAA	80	NM_002468
*TRAF6*	F: GGGAACGATACGCCTTACAA R: CTCTGTCTTAGGGCGTCCAG	174	NM_001105286.1
*NF-κB*	F: CTCGCACAAGGAGACATGAA R: ACTCAGCCGGAAGGCATTAT	147	NM_001048232.1
*β-actin*	F: GGACTTCGAGCAGGAGATGG R: AGGAAGGAGGGCTGGAAGAG	138	XM_021086047.1

*F, forward; R, reverse; ZO-1, zona occluden-1; MUC-2, mucin-2; TLR4, Toll-like receptor 4; MyD88, myeloid differentiation factor 88; TRAF6, tumor necrosis factor receptor-associated factor 6; NF-κB, nuclear factor-kappa B.*

### Western Blot Analysis

According to the described method (Zhou et al., 2020), the total proteins of duodenal tissue were extracted by radio immunoprecipitation assay (Beyotime, Shanghai, China). The protein concentrations were quantified by using the BCA protein assay kit (CoWin Biosciences, Beijing, China) through the BCA method with bovine serum albumin as a standard. Next, the proteins were separated and transferred by sodium dodecyl sulfate polyacrylamide gel electrophoresis gel and polyvinylidenedifluoride membranes (Millipore, Boston, MA) and probed with primary antibodies overnight. The antibodies included zona occluden-1 (ZO-1, 13663, Cell Signaling Technology, United States), occludin (ab167161, Abcam, United Kingdom), claudin-1 (ab180158, Abcam, United Kingdom), TLR4 (ab22048, Abcam, United Kingdom), myeloid differentiation factor 88 (MyD88, 4283, Cell Signaling Technology, United States), tumor necrosis factor receptor-associated factor 6 (TRAF6, ab33915, Abcam, United Kingdom), inhibitor of nuclear factor-kappa B alpha (IκB-α, ab32518, Abcam, United Kingdom), phosphorylation of inhibitor of nuclear factor-kappa B alpha (p-IκB-α, 5209, Cell Signaling Technology, United States), nuclear factor-kappa B p65 (NF-κB p65, 10745-1-AP, Proteintech, United States), phosphor-nuclear factor-kappa B p65 (p-NF-κB p65, 3039, Cell Signaling Technology, United States), and β-tubulin (SungeneBiotech, Tianjin, China). The membranes were washed extensively and incubated with the appropriate secondary antibodies (SungeneBiotech, Tianjin, China) for 1 h. Finally, the immunoreactive bands were detected using ChemiDOC™XRS^+^ and the Image Lab™ System (Bio-Rad).

### 16S rRNA Sequencing of Colonic Microbiota

Total genomic DNA of bacteria in the colonic digesta was extracted from each sample using the cetyltrimethylammonium bromide and sodium dodecyl sulfate method. Illumina NovSeq sequencing and general data analyses were performed by Novogene Biological Information Technology Co. (Beijing, China). The detailed methods have been described previously (Lu et al., 2019). The DNA (regions V3 and V4 of the bacterial 16S rRNA gene) was amplified with barcoded specific bacterial primers using PCR. The primers used in the present study were 341 F: 5’-CCTAYGGGRBGCASCAG-3’ and 806 R: 5’-GGACTACNNGGGTATCTAAT-3’. Purified amplicons were pooled in equimolar and 250 paired-end reads on an Illumina NOVSeq platform.

### Bioinformatics Analysis

The details of the bioinformatics analysis were described in a previous study (Lu et al., 2019). Briefly, QIIME (Version 1.9.1)^1^ was used to demultiplex and filter the original microbial sequencing data. Then, the high-quality sequences were grouped into operational taxonomy units (OTUs) and clustered with a 97% similarity cutoff using UPARSE (version 7.0.1001).^2^ Finally, the ribosomal database project was applied to classify the OTU sequences and to identify the bacterial taxonomy. Alpha diversity was measured by calculating the diversity indexes of Chao1, ACE, Shannon and Simpson. Beta diversity was analyzed by calculating principal component analysis (PCA). Alpha diversity and beta diversity were all determined by QIIME (Version 1.9.1) and R software (Version 2.15.3). The relative abundances at the phylum, family, and genus levels were also analyzed. The linear discriminant analysis effect size (LEfSe) method was applied to compare and to visualize different taxa microbes among groups based on the nonparametric factorial Kruskal-Wallis sum-rank test.

### Short-Chain Fatty Acids Analysis

Determination of SCFAs concentrations with gas chromatography was conducted according to a previous study (Berni Canani et al., 2016). Briefly, 0.3 mL 25 (w/v) metaphosphoric acid was added to 1.5 mL supernatant of colonic digesta to isolate protein and impurities. Next, 0.2 mL crotonic acid was added to 1 mL supernatant for 1 h at 5°C, and then the mixture was injected into a gas phase bottle and loaded onto an Agilent Technologies (Santa Clara, CA, United States) 7820A gas chromatograph system with an automatic loader/injector. The FID detector and AE FFAP capillary column (30 m × 0.25 mm × 0.33 μm fused silica column; ATECH Technologies Co., Ltd., Lanzhou, China) were used.

### Statistical Analysis

Data were analyzed by *t*-tests (and nonparametric tests) using GraphPad Prism 8.0.1 (GraphPad Software, San Diego, CA, United States). The results are expressed as the mean ± standard error of the mean (SEM), and *P* < 0.05 was considered significant (*P < 0.05; ^**^P < 0.01).

## Results

### Effects of *Cordyceps militaris* on Growth Performance in Pigs

To evaluate the effect of the CM on growth performance, pigs were fed a control diet or CM diet for a period of 88 days. Growth performance (Table 4), including the average daily feed intake, final body weight, average daily gain, and feed-to-gain ratio, were not significantly different between the control group and CM group (*P* > 0.05). Collectively, these findings showed that CM had no adverse effect on growth performance in pigs.

**TABLE 4:** Effect of CM on growth performance of pigs.

Item	Control	CM	*P*-value
Initial body weight (kg)	36.88 ± 0.20	36.78 ± 0.21	0.486
Final body weight (kg)	117.16 ± 5.38	120.36 ± 1.29	0.289
Average daily gain (kg/d)	0.93 ± 0.06	0.97 ± 0.01	0.276
Average daily feed intake (kg/d)	2.33 ± 0.36	2.61 ± 0.03	0.258
Feed : Gain	2.67 ± 0.05	2.62 ± 0.05	0.274

*Control, pigs fed a basal diet; CM, pigs fed the basal diet supplemented with CM at 300 mg/kg. Data are presented as the mean ± SEM (n = 5).*

### *Cordyceps militaris* Improves Duodenal Morphology and Physical Barrier Function

**FIGURE 1:**
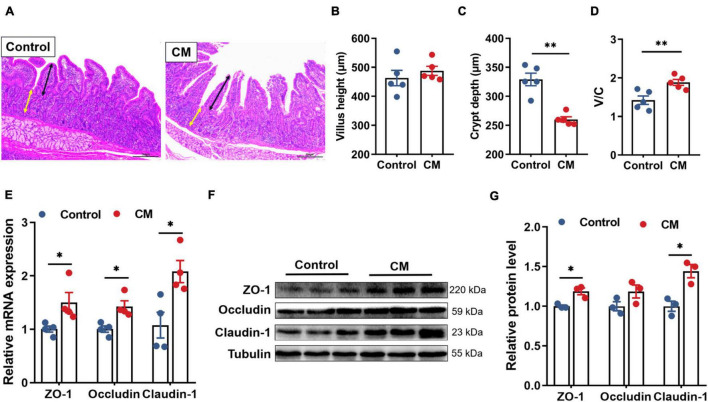
CM improves duodenal morphology and physical barrier function. **(A)** HE-stained section of duodenum, **_↔_** (black) represents the height of villi, **_↔_** (yellow) represents the depth of crypts. **(B)** The villus height (*n* = 5). **(C)** The crypt depth (*n* = 5). **(D)** The ratio of the villus height and crypt dept (*n* = 5). **(E)** RT-qPCR analysis of tight junction proteins ZO-1, occludin, and cladin-1 (*n* = 4). **(F,G)** Western blot analysis of tight junction proteins ZO-1, occludin, and cladin-1 (*n* = 3). Control, pigs fed a basal diet; CM, pigs fed the basal diet supplemented with CM at 300 mg/kg. Data are presented as the mean ± SEM. *P < 0.05, ^**^P < 0.01.

We next performed H&E staining on the duodenum to investigate the effect of the CM on duodenal morphology (Figure 1A). By measuring the villus height and the crypt depth (Figures 1B–D), it was found that the CM group did not affect the villus height (*P* > 0.05), but it could significantly decrease the crypt depth and increase the ratio of villus height and crypt depth (V/C) (*P* < 0.01). In addition, we assessed the effect of the CM on the intestinal physical barrier by detecting the expression of TJPs. The results of RT-qPCR (Figure 1E) showed that the CM group significantly elevated the mRNA expression levels of ZO-1, occludin, and claudin-1 (*P* < 0.05). Similarly, the Western blot results (Figures 1F,G) showed that the CM group also significantly upregulated the protein expression of ZO-1 and claudin-1 (*P* < 0.05).

### *Cordyceps militaris* Improves Biochemical Barrier Function

**FIGURE 2:**
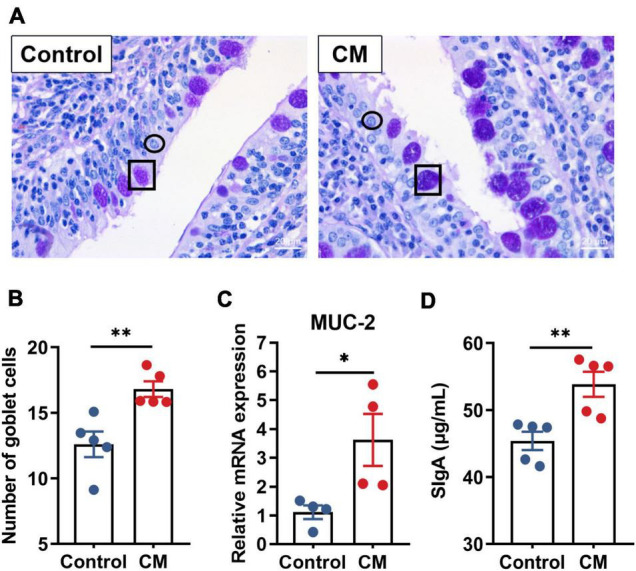
CM improves biochemical barrier function. **(A)** Periodic acid Schiff (PAS) staining of the duodenum, ↔ represents goblet cells, and ↔ represents intestinal epithelial cells. **(B)** Count and analysis of the number of goblet cells (*n* = 5). **(C)** RT-qPCR analysis of the mRNA expression level of *MUC-2* (*n* = 4). **(D)** ELISA analysis of the concentration of SIgA in the duodenal mucosa (*n* = 5). Control, pigs fed a basal diet; CM, pigs fed the basal diet supplemented with CM at 300 mg/kg. Data are presented as the mean ± SEM. *P < 0.05, ^**^P < 0.01.

To explore the effect of the CM on biochemical barrier function, we performed PAS staining on the duodenum (Figure 2A) and detected the expression level of mucin-2 (*MUC-2*) and the concentration of SIgA. After counting the number of goblet cells (Figure 2B), compared to the control group, we found that the CM group significantly increased the number of goblet cells (*P* < 0.01). The RT-qPCR results (Figure 2C) revealed that the CM group also remarkably upregulated the mRNA expression level of *MUC-2* secreted mainly by goblet cells (*P* < 0.05). Furthermore, the CM group also significantly increased the concentration of SIgA (Figure 2D) in the mucosa (*P* < 0.01).

### *Cordyceps militaris* Improves Immunological Barrier Function

**FIGURE 3:**
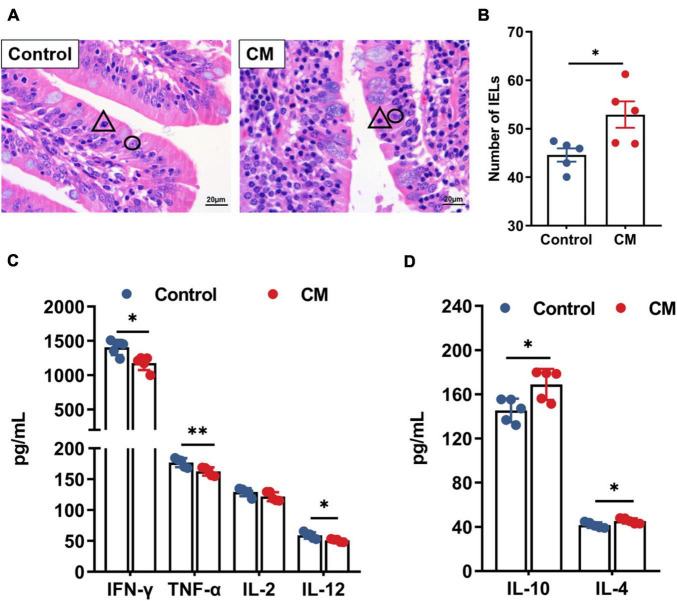
CM improves immunological barrier function. **(A)** Hematoxylin-eosin stained sections of the duodenum, △ represents intestinal intraepithelial lymphocytes, and ° represents intestinal epithelial cells. **(B)** The number of intraepithelial lymphocytes was counted and analyzed. **(C,D)** ELISA analysis of the levels of pro-inflammatory cytokines and anti-inflammatory cytokines in the duodenal mucosa. Control, pigs fed a basal diet; CM, pigs fed the basal diet supplemented with CM at 300 mg/kg. Data are presented as the mean ± SEM (*n* = 5). *P < 0.05, ^**^P < 0.01.

Since immunological barrier function is closely related to IELs and cytokines, we counted the number of IELs (Figures 3A,B) and examined the mucosal levels of cytokines (Figures 3C,D). The results showed that the number of IELs was significantly higher in the CM group than in the control group (*P* < 0.05). Moreover, the CM group reduced the levels of the proinflammatory cytokines IFN-γ (*P* < 0.05), TNF-α (*P* < 0.01), and IL-12 (*P* < 0.05), while it elevated the levels of the anti-inflammatory cytokines IL-10 and IL-4 (*P* < 0.05).

### *Cordyceps militaris* Inhibits the TLR4/MyD88/NF-κB Signaling Pathway

**FIGURE 4:**
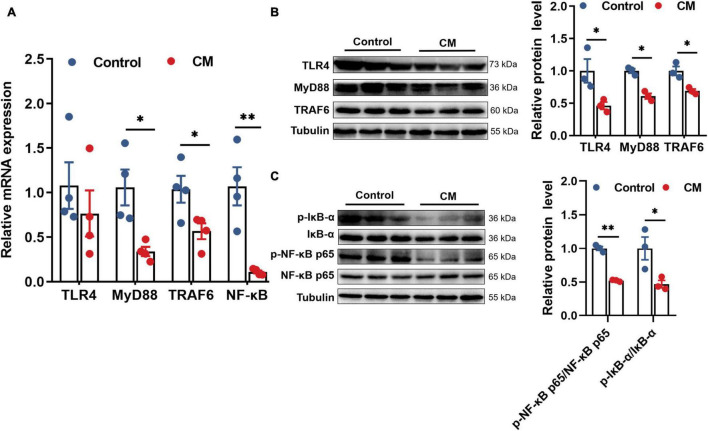
CM inhibits the TLR4/MyD88/NF-κB signaling pathway. **(A)** RT-qPCR analysis of the mRNA expression levels of TLR4, MyD88, TRAF6, and NF-κB (*n* = 4). **(B)** Western blot analysis of the protein expression levels of TLR4, MyD88, and TRAF6 (*n* = 3). **(C)** Western blot analysis of the protein expression levels of p-NF-κB p65 and p-IκB-α (*n* = 3). Control, pigs fed a basal diet; CM, pigs fed the basal diet supplemented with CM at 300 mg/kg. Data are presented as the mean ± SEM. *P < 0.05, ^**^P < 0.01.

Based on the changes in the levels of cytokines, the expression of certain cytokines is regulated by the prototypical TLR4/NF-κB signaling pathway. Thus, we examined the TLR4/NF-κB signaling pathway to explore the mechanisms by which CM regulates cytokines. RT-qPCR analysis showed that the gene expression levels of MyD88 (*P* < 0.05), TRAF6 (*P* < 0.05) and NF-κB (*P* < 0.01) in the CM group were significantly decreased (Figure 4A). Based on the gene expression results, we further assessed the protein expression of the TLR4/MyD88/NF-κB pathway by Western blot. As shown in Figures 4B,C, the CM group remarkably downregulated the protein expression of TLR4 (*P* < 0.05), MyD88 (*P* < 0.05), TRAF6 (*P* < 0.05), p-IκB-α (*P* < 0.05), and p-NF-κB p65 (*P* < 0.01). In summary, CM inhibited the TLR4/MyD88/NF-κB signaling pathway.

### *Cordyceps militaris* Changes the Composition of Colonic Microbiota

**FIGURE 5:**
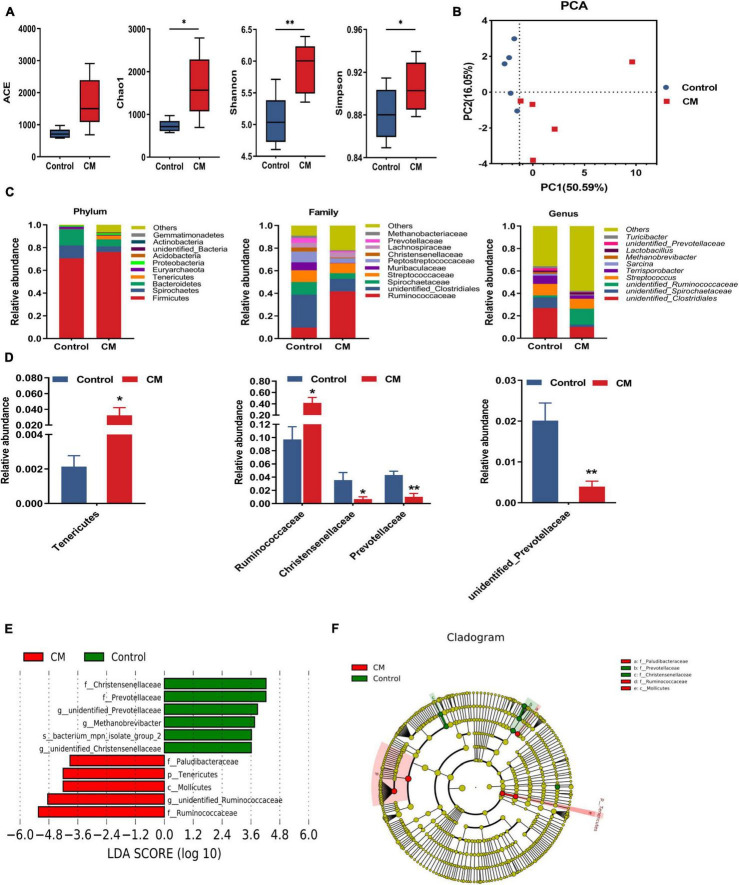
CM changes the composition of the colonic microbiota. **(A)** Alpha diversity indexes (Ace, Chao1, Shannon, and Simpson) of colonic microbiota. **(B)** The principal component analysis (PCA) of colonic microbiota. **(C)** Microbiota compositions at the phylum level, family level, and genus level. **(D)** Statistical analysis of the differences in microbiota at the phylum level, family level, and genus level. **(E)** LDA score. Enriched taxa with an LDA score > 3.6 are shown in the histogram, control group-enriched taxa are indicated with a positive LDA score (green), and the taxa enriched by the CM group have a negative score (red). **(F)** LEfSe taxonomic cladogram. Taxa enriched in the CM group are shown in red, and taxa enriched in the Control group are shown in green. Control, pigs fed a basal diet; CM, pigs fed the basal diet supplemented with CM at 300 mg/kg. Data are presented as the mean ± SEM (*n* = 5). *P < 0.05, **P < 0.01.

To understand the changes in the microbial community in the colon, we used a high-throughput sequencing method based on the 16S rRNA genes to analyze the effect of CM on colonic microbiota. An average of 85,194 raw reads were generated from each sample. After removing the low-quality sequences, 62,252 effective tags were clustered into OTUs. A total of 7,748 OTUs were obtained by clustering with 97% identity. To evaluate the difference in colonic microbiota between the two groups, we conducted alpha diversity analysis and beta diversity analysis. The richness of the microbial community was evaluated by measuring the ACE and Chao1 indexes, and the diversity and uniformity of the microbial community were reflected by the Shannon and Simpson indexes. As shown in Figure 5A, the Chao1 (*P* < 0.05), Shannon (*P* < 0.01), and Simpson (*P* < 0.05) indexes in the CM group were significantly higher than those in the control group. Furthermore, we analyzed the distinct microbiota composition between the two groups by analyzing PCA. As shown in Figure 5B, the two principal components account for 50.59 and 16.05% of the total variance, respectively. The control group and CM group exhibited a distinct clustering of microbiota composition.

After analyzing the intestinal microbial composition at different levels, it was found that the CM group changed the microbial composition of the colon. As shown in Figures 5C,D, the different microbial compositions of the two groups were displayed at the phylum, family, and genus levels. At the phylum level, the dominant bacteria between the two groups were *Firmicutes, Spirochaetes, Bacteroidetes*, and *Tenericutes* (average relative abundance ≥ 1%). Compared with the control group, the CM group showed a higher relative abundance of *Tenericutes* (*P* < 0.05). At the family level, the relative abundance of *Ruminococcaceae* was significantly increased (*P* < 0.05), but *Christensellercae* (*P* < 0.05) and *Prevotellaceae* (*P* < 0.01) were significantly decreased in the CM group. At the genus level, the CM group significantly reduced the relative abundance of *unidentified Prevotellaceae* (*P* < 0.01). To further identify the alteration in the microbial structure, we compared the colonic microbiota of the two groups using the LEfSe method. Linear discriminant analysis (LDA) results showed that the CM group enriched the abundance of *Ruminococcaceae, Mollicutes*, *Tenericutes*, and *Paludibacteraceae*, and the control group enriched the abundance of *Christensellercae, Prevotellaceae, Methanobrevibacter, and bacterium_mpn-isolate_group_2* (Figure 5E). A cladogram representative of the structure of the colonic microbiota and the predominant bacteria is shown in Figure 5F. *Ruminococcaceae* and *Mollicutes* had the highest abundance in the red parts in the CM group, but *Christensellercae* and *Prevotellaceae* were the richest in the green area in the control group. Overall, these findings suggested that the CM group altered the composition of the colonic microbiota and promoted the multiplication of specific bacteria.

### *Cordyceps militaris* Promotes the Production of Short-Chain Fatty Acids in the Colonic Digesta

**FIGURE 6:**
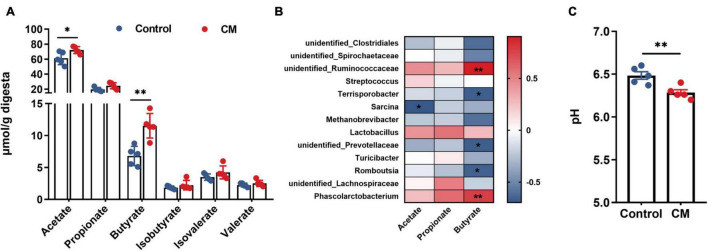
CM promotes the production of SCFAs in the colonic digesta. **(A)** Concentrations of microbial fermentation metabolite SCFAs. **(B)** Spearman’s correlation test was used to analyze the correlation between colonic microbial composition related to SCFAs (acetate, propionate, and butyrate); red represents a positive correlation, and blue represents a negative correlation. **(C)** The pH value in the colonic digesta. Control, pigs fed a basal diet; CM, pigs fed the basal diet supplemented with CM at 300 mg/kg. Data are presented as the mean ± SEM (*n* = 5). *P < 0.05, **P < 0.01.

To further evaluate the effect of CM on microbial metabolites, we measured the concentrations of acetate, propionate, butyrate, isobutyrate, valerate, and isovalerate in the colonic digesta. As shown in Figure 6A, the CM group significantly increased the concentrations of acetate (*P* < 0.05) and butyrate (*P* < 0.01). However, the concentrations of propionate, isobutyrate, valerate, and isovalerate were not significantly different between the CM group and the control group (*P* > 0.05). Because the production of SCFAs was highly associated with gut microbiota, we used Spearman’s correlation test to analyze the correlation between SCFAs and colonic microbiota. As shown in Figure 6B, acetate was negatively associated with the relative abundance of *Sarcina.* Butyrate was positively associated with the relative abundances of *unidentified Ruminococcaceae*, and *Phascolarctobacterium* but negatively correlated with the relative abundances of *Terrisporobacter, unidentified Prevotellaceae, and Romboutsia.* In addition, the data showed that the pH value (Figure 6C) of the CM group was significantly lower than that of the control group in the colon (*P* < 0.01).

## Discussion

The beneficial effects of CM on intestinal microbiota and immune activity have been widely discussed using mice or *in vitro* models (Jo et al., 2010; Fan et al., 2018; Gamage et al., 2018), which are more or less unable to well convey the scenario in humans. Here, pigs, whose anatomical and physiological characters are much closer to human beings, were employed to explore the effects of CM on intestinal mucosal barrier function and colonic microbiota.

Intestinal villi and crypts directly affect the intestinal epithelial barrier and absorption function (Gehart and Clevers, 2019). In this study, CM significantly decreased the crypt depth and significantly raised V/C in the duodenum, indicating that CM benefits duodenal function. Besides, CM upregulated the expression of ZO-1 and claudin-1 in the duodenal mucosa, which could maintain intestinal barrier integrity and regulate intestinal permeability (Kurashima and Kiyono, 2017), for down regulation of the expression of ZO-1 and claudin-1 increased intestinal permeability and caused intestinal-related diseases (Piche et al., 2009; Ahmad et al., 2017). Collectively, intake of CM improved intestinal physical barrier.

Mucins and SIgA covering the intestinal epithelium serve as an important part of the biochemical barrier to protect the intestine (Kurashima and Kiyono, 2017). The mucins and enterocytes provide the first defense line of the gastrointestinal tract and interact with the immune system (Pelaseyed et al., 2014). Here, CM significantly increased the number of goblet cells and elevated the expression levels of MUC-2 and SIgA, which fit well with previous studies, reporting that MUC-2 served as an important structural component of the intestinal mucin layer, which protected against the invasion of luminal virus, and its absence affected the repair of mucosa (Wallace et al., 2011; Pelaseyed et al., 2014). Furthermore, SIgA contributes as a first-line barrier that protects the epithelium from pathogens and toxins (Pabst, 2012). In short, CM enhanced the defense function of the intestinal biochemical barrier.

Intestinal mucosal immunity promotes the protective immune response against pathogens through the anti-inflammatory and pro-inflammatory responses exerted by cytokines (Perez-Lopez et al., 2016). Our results suggested that CM significantly upregulated the levels of the anti-inflammatory cytokines IL-10 and IL-4 but downregulated the pro-inflammatory cytokines INF-γ, TNF-α, and IL-12. IL-12 induces T helper 1 (Th1) cell differentiation, which secretes IFN-γ, TNF-α, and IL-2 (Wong et al., 2011), and IL-4 induces T helper 2 (Th2) cell differentiation, which secretes IL-4, IL-5, and IL-10 (Zhu et al., 2010). Furthermore, cytokines also affect the integrity of the intestinal barrier (Pu et al., 2020). Proinflammatory cytokines (such as TNF-α) could increase the tissue permeability of luminal antigens by disrupting tight junctions, while anti-inflammatory cytokines (such as IL-10) tend to protect the integrity of the intestinal barrier (Al-Sadi et al., 2009; Pu et al., 2020). Similarly, previous studies have also shown that CM decreased the activity of TNF-α, IL-12, and INF-γ in humans (Sun et al., 2014) and effectively recovered cyclophosphamide-induced decreases in IL-10 levels (Shin et al., 2018). A recent study also found that *Cordyceps sinensis* polysaccharides also stimulated TNF-α, IL-12, and INF-γ secretion in cyclophosphamide-treated mice (Ying et al., 2020). Interestingly, the cytokines IL-4 and IL-10 secreted by Th2 could promote the mucosal IgA response (Mantis et al., 2011), which is consistent with our results. Therefore, CM may improve intestinal barrier function by enhancing the synergy of the biochemical barrier and immunological barrier. These findings indicated that CM might regulate intestinal mucosal immunity and protect the integrity of the intestinal barrier.

Growing evidence suggests that TLR4 plays an important role in autoimmunological disease, and it is best known for recognizing LPS and leading NF-κB nuclear translocation *via* the signaling pathway containing MyD88, IRAK1, and TRAF6 (Liu et al., 2012; Plociennikowska et al., 2015). NF-κB is an important transcription factor in the regulation of pro-inflammatory cytokines (Lawrence, 2009). Therefore, we further explored the mechanism by which CM regulates inflammation. Here, CM inhibited the expression of the TLR4/MyD88/NF-κB signaling pathway, which was in consistence with a previous report showing that CM fruit body extract relieved nephritis by inhibiting the TLR4/NF-κB signaling pathway (Song et al., 2016). Additionally, polysaccharides, as one of the main active components of CM, was documented to protect against immunosuppression *via* TLR4/NF-κB signaling pathway in mice (Meng et al., 2019). Overall, it’s concluded that CM regulates inflammation *via* inhibiting the TLR4/MyD88/NF-κB signaling pathway.

Overwhelming evidence has demonstrated that the gut microbiota and its metabolites are essential for intestinal function and health (Sekirov et al., 2010; Donaldson et al., 2016). CM increased the diversity of colonic microbiota, indicating that CM enhanced resistance to intestinal diseases, since the decrease in the diversity of the microbiota is associated with ulcerative colitis (UC) or Crohn’s disease (Allen et al., 2015). Moreover, CM also changed the composition of the colonic microbiota. CM increased the abundance of gram-positive bacteria (*Ruminococcaceae*) and decreased the abundance of gram-negative bacteria (*Prevotellaceae*), which may partially explain why CM inhibited the TLR4/MyD88/NF-κB signaling pathway induced by LPS. *Ruminococcaceae* has been fully proven to be the cause of the degradation of various polysaccharides and fibers (Hooda et al., 2012), and *Ruminococcaceae* is inversely correlated with UC and inflammatory bowel disease (Zhai et al., 2019; Sinha et al., 2020). Moreover, *Ruminococcaceae* is a major producer of butyrate (Louis and Flint, 2017), and the correlation between *Ruminococcaceae* and butyrate was also observed in our correlation analysis. On the other side, *Prevotellaceae* was positively related to colorectal cancer (Yu, 2018; Ibrahim et al., 2019), which seriously damaged intestinal barrier function. Thus, CM rich in cordyceps polysaccharide boosts intestinal health partially through optimizing the composition of intestinal microbiota.

Intriguingly, cordycepin in present study dramatically recovered the relative abundance of *Bacteroidetes* and *Firmicutes*, which were more abundant in obese rats (An et al., 2018), Within our knowledge, there is no convincing explanation yet about the obscure link between improved health and elevated ratio of *Bacteroidetes* to *Firmicutes*. We supposed that the adverse effects of elevated ratio of *Bacteroidetes* to *Firmicutes*, if any, might be covered by the accumulated benefits resulted from the other gram-positive bacteria, *Ruminococcaceae* or as described above. However, in this study, CM increased the relative abundance of *Tenericutes*, which may be caused by different species of experimental animals, but the specific reasons require further research for evaluation. It is worth noting that most of the bacteria reduced in the CM group are potentially associated with intestinal health. The human intestinal bacteria *Christensenella* are widespread, heritable, and related to health (Waters and Ley, 2019), but there are few related studies on pigs. *Methanobrevibacter* has been shown to be indirectly related to severe diseases of the colon (Chaudhary et al., 2018).

SCFAs are well known to exert important role in immunity, and intestinal function (Koh et al., 2016). Of note, acetate and butyrate may modulate immunity, inflammation, intestinal integrity and regulate transcription by way of epigenetic mechanisms (Liu et al., 2018; Neu and Pammi, 2018). In this study, CM increased the production of acetate and butyrate, which was similar with cordyceps polysaccharides reported previously (Fan et al., 2018; Ying et al., 2020). In addition, CM decreased the pH value in the colon of pigs, which fits well with a previous report, where an increase in SCFAs may lead to a decrease in the pH value, and a lower pH value may result in reduced bacterial or viral populations (Sun et al., 2015). Thus, SCFAs might be an important mediator of the beneficial effects of CM to intestinal health.

SCFAs, metabolites of intestinal microbiota, strengthen mucosal barriers through multiple pathways (Morrison and Preston, 2016; Wan et al., 2020). Firstly, SCFAs play an essential role in regulating the integrity of the physical barrier through the cooperation with TJPs (Yan and Ajuwon, 2017; Zheng et al., 2017). Secondly, SCFAs have been demonstrated to upregulate the expression of mucin-related genes to improve the biochemical barrier (Willemsen et al., 2003; Gaudier et al., 2004). A recent study found that a reduction in SCFAs exacerbated intestinal permeability by downregulating the expression of mucin and TJPs (Liu et al., 2020). Thirdly, SCFAs can also inhibit inflammation and downregulate the production of proinflammatory cytokines to enhance intestinal immunological barrier function (Morrison and Preston, 2016; Liu et al., 2019). Therefore, we assumed that the intestinal mucosal barrier function might be regulated by the SCFAs produced by the microbiota.

## Conclusion

**FIGURE 7:**
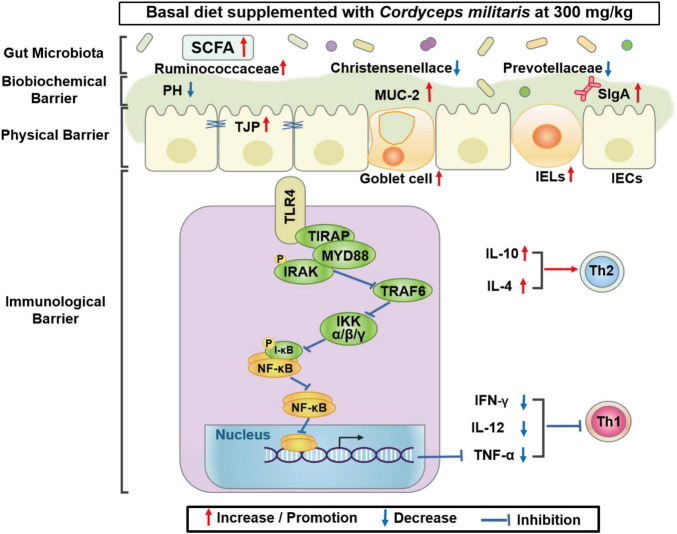
Proposed model of the effects of CM on intestinal barrier function and gut microbiota of pigs. Diagram showing the effects of CM on the intestinal physical barrier, biochemical barrier, immunological barrier, and gut microbiota. Mechanistically, CM may inhibit inflammation *via* the TLR4/MyD88/NF-κB signaling pathway.

In conclusion, CM enhanced intestinal barrier function, indicated by improved physical barrier, biochemical barrier, and immunological barrier function in pigs. Mechanistically, CM repressed inflammation by inhibiting the TLR4/MyD88/NF-κB signaling pathway. Furthermore, CM altered the composition of the colonic microbiota and increased the concentration of SCFAs in pigs (Figure 7). This study finds a novel role for CM in the modulation of intestinal epithelial barrier function and gut microbiota, which may provide a nutritional strategy to improve intestinal health in farm animals.

## Data Availability Statement

The raw data supporting the conclusions of this article will be made available by the authors, without undue reservation.

## Ethics Statement

The animal study was reviewed and approved by the Animal Welfare Committee of Northwest A&F University (Yangling, Shaanxi, China).

## Author Contributions

XS, HZ, and HC designed the study, wrote, and revised the manuscript. DZ, JH, JSL, SW, and JFL helped took samples and performed the experiments and analyses. HZ, HC, XL, GY, and XS edited the manuscript. All authors have read and agreed to the published version of the manuscript.

## Conflict of Interest

The authors declare that the research was conducted in the absence of any commercial or financial relationships that could be construed as a potential conflict of interest.

## Publisher’s Note

All claims expressed in this article are solely those of the authors and do not necessarily represent those of their affiliated organizations, or those of the publisher, the editors and the reviewers. Any product that may be evaluated in this article, or claim that may be made by its manufacturer, is not guaranteed or endorsed by the publisher.

## Footnotes

1
http://qiime.org/scripts/split_libraries_fastq.html

2
http://drive5.com/uparse/

## Abbreviations

CM: *Cordyceps militaris*
IECs: intestinal epithelial cells
IELs: intraepithelial lymphocytes
H&E: hematoxylin and eosin
PAS: periodic acid schiff
RT-qPCR: real-time quantitative polymerase chain reaction
V/C: the ratio of villus height and crypt depth
TJPs: tight junction proteins
ZO-1: zona occluden-1
GALT: gut-associated lymphoid tissue
MUC-2: mucin-2
ELISA: enzyme-linked immunosorbent assay
SIgA: Secretoryn immunoglobulin A
IgG: immunoglobulin G
IgA: immunoglobulin A
IgM: immunoglobulin M
IFN-γ: interferon- γ
TNF-α: tumor necrosis factor α
IL-10: interleukin-10
IL-4: interleukin-4
IL-2: interleukin-2
IL-12: interleukin-12
Treg: regulatory T
Th: T helper 1
Th2: T helper 2
SCFAs: short-chain fatty acids
TLR4: Toll-like receptor 4
MyD88: myeloid differentiation factor 88
TRAF6: tumor necrosis factor receptor-associated factor 6
IκB-α: inhibitor of nuclear factor-kappa B alpha
p-IκB-α: phosphorylation of inhibitor of nuclear factor-kappa B alpha
NF-κB: nuclear factor-kappa B
p-NF-κB p65: phosphor-nuclear factor-kappa B p65
OTUs: operational taxonomy units
PCA: principal component analysis
LEfSe: linear discriminant analysis effect size
LDA: linear discriminant analysis.
